# Empirical Analysis of Factors Influencing Healthcare Efficiency among Emerging Countries

**DOI:** 10.3390/healthcare9010031

**Published:** 2020-12-31

**Authors:** Lulin Zhou, Sabina Ampon-Wireko, Lamini Dauda, Xinglong Xu, Maxwell Opuni Antwi, Ebenezer Larnyo

**Affiliations:** School of Management Science, Jiangsu University, Zhenjiang 212013, China; zll62@ujs.edu.cn (L.Z.); 5103170212@stmail.ujs.edu.cn (L.D.); 1000004932@ujs.edu.cn (X.X.); 5103170109@stmail.ujs.edu.cn (M.O.A.); dr.ebenlarnyo@stmail.ujs.edu.cn (E.L.)

**Keywords:** healthcare efficiency, health care expenditure, data envelopment analysis, emerging nations

## Abstract

Numerous factors, including inefficient utilization of healthcare resources have been attributed to the poor health outcome. The study aims to compare the efficiency of health expenses and its determining factors in the emerging economies based on their income levels. Data for the study is extracted from the World Bank’s World Development Indicators for 21 countries covering the period of 2000 to 2018. Analysis of the research involves two stages. Stage one computes the efficiency scores, whereas second stage examines factors affecting health efficiency by employing the Tobit regression and Simar-Wilson regression test to confirm the results. The Tobit result shows that research and development (R&D) and physicians enhanced health efficiency at the main panel, lower-middle-income, upper-middle-income, and high-income countries. Corruption remained negative with education showing mixed results. The interaction between research and development and physicians increases health efficiency in all the panels. Health research must be a policy focus if efficiency is to be achieved by the emerging economies.

## 1. Introduction

Improving population health is a subject of great concern worldwide, and this is expected to reflect in actions taken by leaders to ensure access to better healthcare services [1]. For this reason, increasing public spending on health care has become a priority across the globe. Grigoli and Kapsoli [2] asserted that inefficiencies undermine national efforts to strengthen the health systems. The World Health Report (WHR) 2010 estimated that about 20%–40% of health sector resources are wasted [3]. This could lead to several undesirable health consequences, which can affect the general public. In emerging economies, the health system is confronted with unstable health costs such as gaps in safety, quality, access, and equity. The World Health Organization (WHO) [4] established that the decline in expenses in the health sector might not promote better outcomes and equitable use of health resources.

While the scarcity of funds for health exists everywhere, health care efficiency is a comparison of outputs, such as mortality and mobility with inputs, including public human resources, improved health status, financial risk protection, and public satisfaction [3] using Data Envelopment Analysis (DEA) or stochastic frontier analysis (SFA).

Inefficiencies within the health sector occur in diverse ways such as hospital management, admissions, and health worker performance. Over the years, the emerging economies have made remarkable progress to ensure improved health services by decreasing non-communicable diseases and prolonging life. Nevertheless, the epidemiological change with roots cause of death shifting slowly from infectious to chronic diseases puts healthcare systems of emerging countries under financial pressure. As such, the efficiency of financial and human resources employed in the health sectors across emerging economies becomes an essential topic for researchers and decision-makers within the healthcare sector.

Previous research established significant health spending inefficiencies among industrialized economies and other developing countries [5]. Considering the growing economic pressures among emerging economies, policy-makers, systems leaders, private payers, and consumers seek ways to lessen waste, enhance efficiency, and improve the value of healthcare [6]. Several studies such as Wang and Li [7], Liu and Xia [8], Cetin and Bahce [9], Auerbach, [10], Alin and Marieta [11], Eriksen and Wiese [12], Herwartz and Schley [13], Althin, and Färe [14], Barthold andNandi [15], and Allin, Grignon [16] have been carried out. Briefly, this current study differs from the literature on happiness [17], nursing homes and hospitals Wang and Li [7], Scott-Emuakpor [18], Olanubi and Osode [19].

To understand the factors governing inefficiencies in health sector, Eriksen [13] examined Germany’s case by using the stochastic frontier model. A standard mortality rate was employed as an output variable. General practitioners, hospital beds, and the number of specialists were used as input variables. The study found lower efficiencies in urban and higher efficiencies in rural areas. To inform related policy discussions among the European nations, Herwartz, and Schle [4] measures the efficiency and productivity of breast and lung cancer health care expenditure. Input variables such as the number of radiation units, number of oncologists, and oncology pharmaceuticals were used to produce survival and quality of life. The findings from their study revealed efficient and inefficient health resources among both wealthy and less affluent countries. Moreover, among the 34 organizations for economic co-operation and development (OECD) countries, Cetin and Bahçe, [9] examined the efficiency of health systems using the decision-making units (DMUs) such as the number of doctors, number of patient beds, and health expenditure per capita were used as input variables. Life expectancy at birth and infant mortality rate were used as outputs. Their study showed that countries producing good health services with fewer inputs such as Chile, Mexico, and Turkey are reference countries for others with much better outputs.

In the case of Nigeria, Olanubi, and Osode, [19] examined the efficiency of public funds allocated to human resources for health during six government regimes for 1966–2014 by utilizing the stochastic frontier analysis. Similarly, Tormusa and Idom, [20] studied the impediments of corruption on the efficiency of delivering healthcare services in Nigeria and concluded that corruption threatens health care access, fairness, and outcomes and should, therefore, be taken into serious consideration as the less privileged in the society suffers most. Based on the findings of other studies, Stefko, Gavurova et al. [21] covers healthcare resources’ efficiency in Slovak Republic for the period 2008–2015. Data envelop analysis was used with two stable inputs (number of beds, number of medical staff) and other inputs such as the number of all medical equipment, magnetic resonance devices, and computed tomography devices. The study disclosed that regions with lower values of variables over time achieved a high degree of efficiency and vice-versa.

Ibrahim and Daneshvar [22] utilized the DEA to study the healthcare system’s efficiency in Lebanon. Life expectancy at birth, maternal mortality ratio, infant mortality rate, and people newly infected with human immune virus were used as output, whereas public healthcare expenditure and hospital beds served as an input variable. Using the Data Envelopment Analysis (DEA) technique developed by Charnels et al. [23], it was found that the healthcare system in Lebanon continuously enhanced its efficiency between the period 2000 and 2015. Gearhart and Michieka [24] studied the role of natural resource abundance on healthcare efficiency in Appalachia with the non-parametric robust order-m estimator for 2012 to 2016 among 420 counties. 

Grigoli and Kapsoli [2] examined the efficiency of health costs in emerging countries by utilizing the stochastic frontier analysis. Their study disclosed that African countries displayed economies with less efficiency. Liu and Xia [8] considered health cost efficiency within rural China by employing the Malmquist productivity index and the super-SBM model. Health cost-efficiency values exhibited unstable trends during the study period. In other countries, studies, such as Cetin and Bahce [9], measured health efficiency. The study concluded that 11 of the 26 countries were health efficient and found room for efficiency development in health systems among the remaining 15 countries. Auerbach, Weeks [10] examined health expenses efficiency among the Veterans Affairs of the United States (U.S.) Department. The study found a suggestive confirmation of health inefficiency principally in the sector of inpatient care. Althin, Färe [14] investigated the efficiency of lung and breast cancer health services in Europe. When analyzing whether nations are health-efficient in terms of improving long life among men and women for OECD nations. Barthold and Nandi [15] used the multivariable regression models and their study revealed health expenses increases were connected with a surge in longevity improvements among men than women. Allin and Grignon [16] carried out an empirical analysis of factors affecting health efficiency in Canada. Their study showed that inefficiencies in the health sector resulted from certain factors such as health management and re-admissions in public health policies.

Literature regarding the efficiency of health expenses at the macro level in emerging economies is still at the elementary stage. Conclusions drawn from the existing literature at micro levels such as See and Yen [17] in nursing homes and hospitals may not be reflecting the accurate picture of health efficiency at the macro level [18,19,25]. As such, questions about reducing wastage of resources in the health sector have increased and, therefore, examining the productive utilization of health resources among emerging countries has become paramount. Are emerging countries efficiently using their health resources? Are there factors affecting the efficiency of health costs in emerging countries? Is there an interplay between corruption and health efficiency in emerging economies?

To answer some of these questions, the researchers take up the challenge of investigating the efficiency of health and its determining factors in the emerging nations by grouping the countries under different income levels. Findings from the study will contribute immensely to filling the current gap in the literature about health efficiency at the macro level while providing essential information to enrich financial health management and programs. Although studies of this nature are vital, previous studies [6,8,9,15,16,22,26] only examined healthcare efficiency without investigating the determining factors, and this could result in misleading inferences for policy implication.

To resolve these discrepancies, the second stage of our study examines the influencing factors of health care efficiency in the emerging economies based on different income groups by incorporating corruption and health research and development (R&D) into the model. In so doing, the study applied the econometric approach by using Tobit regression and a Simar and Wilson regression test for a robust check. The current study further explores the interaction effect between health R&D and physicians in these countries. This is very significant because the present study’s outcome will provide a conceptual understanding of how the presence of these factors work together to enhance healthcare efficiency within the emerging nations. The remaining section of the study is outlined below. Section two presents the method and data employed in the study, while section three gives the analysis of the results. Section four presents the discussion. The last section provides a conclusion and policy recommendation followed by limitation and future direction of this work.

## 2. Materials and Methods

This study employs the data of 21 emerging countries for 2000–2018. The countries include India, Pakistan, Philippines, Bangladesh, Argentina, Brazil, Colombia, Peru, Thailand, China, Venezuela, South Africa, Czech Republic, Russian Federation, Poland, Ukraine, Greece, Turkey, United Arab Emirates, Bulgaria, and Malaysia. The rest of the countries were not included in the study due to the non-availability of data. No ethical approval was required for this study because it was based on secondary analysis of data obtained from the World Bank Development Indicators and the World Health Organization database.

Consistent with World Bank, the study classified the emerging nations further into three sub-groups: high-income, upper-middle, and lower-income countries to obtain more in-depth analysis (Table 1).

### 2.1. Input and Output Variables

A draft of the variable list was prepared after a comprehensive literature review. The list was finalized by checking the availability of data from the World Bank development indicators’ database. Public healthcare expenditure was chosen as an input variable to health production while life expectancy and infant survival rate were selected as output variables. This was based on the fact that public healthcare expenditure has been considered one of the primary indicators of a country’s healthcare system efficiency [2,9]. Health output is latent in nature and, therefore, difficult to quantify directly. To approximate population health indirectly, we considered life expectancy at birth as a robust healthcare system outcome used widely in the literature [27,28,29]. In addition, the inverse infant mortality rate ‘infant survival rate’ as a desirable output following relevant literature of Lionel, [30].

A significant assumption for the DEA model employed in the study was that the selected health status in the emerging nations is dependent on the inputs from healthcare expenditure. The study chose input parameters as proxies for the amount of fee a country devotes to healthcare and child survival rate as output indicators. If infant death rate (*IMR*) is estimated as [(number of children dying before reaching 1 year)/(number of births in a year)] × 1000. The formula used for computing infant survival rate according to Allin and Grignon, [31] is presented as:

(1) $$ISR-\frac{\left(1000-IMR\right)}{IMR}$$

**Figure 1:**
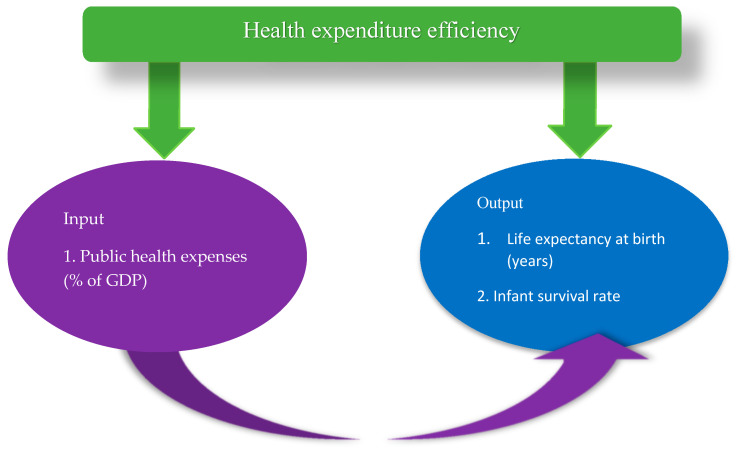
Inputs and output indicators of health cost efficiency among emerging economies. Source: Authors computation 2020.

However, the health outcome variables are extracted from the World Development Indicators. The association between health cost and health status considered in this study is in line with the view that increasing longevity and reducing child mortality signifies improving a country’s health outcome. Following the model, it would be expected that the higher the public health expenditure, the greater the life expectancy and the ratio of young children who survived the first year. Following Cetin and Bahce [9] in OECD and Alin and Marieta [11] in the European Union, the variable represented in Figure 1 is used as input and output indicators for health efficiency in emerging economies.

### 2.2. Variable Selection Criteria

Recent studies on the determinants of healthcare efficiency showed that human resource factors such as the number of physicians have been a source of efficiency in health systems [3,24,31]. Other studies [17,32] disclosed that government quality (control of corruption) has a massive challenge for achieving health goals in Nigeria. Understanding the mechanism by which education affects health is, hence, crucial for policy. A major relationship between education and health has been established and observed in many countries for a wider variety of health measures [33]. Cochrane, O’Hara [34] disclosed a significant relationship between education and health. Grossman [35] also asserted that education could improve health by raising efficiency in health production. More educated people tend to be knowledgeable and make better choice with regards to health issues [36]. Social-economic factors such as GDP per capita have some effect on health efficiency to some large extent [19]. According to Knottnerus [37], the development of medical research offers opportunities to meet these health challenges by coming out with better ways to treat, prevent diseases, and improve lives. Medical research has demonstrated its value over the long term by providing interventions to conditions like polio, which is on the verge of global eradication.

### 2.3. Definition of Variables

Corruption (CRP) reflects perceptions of the extent to which public power is exercised for private gain, including both petty and grand forms of corruption. Public health expenditure (PHCE) refers to funds released by governments for medical care, prevention, promotion, rehabilitation, community health activities, health administration, and capital formation with the predominant objective of improving health. Infant survival rate (ISR) is defined as the ratio of children that survived the first year of life.

Life expectancy at birth (LER) indicates the number of years a newborn infant would live if prevailing patterns of mortality at the time of its birth were to stay the same throughout its life.

Research and development (R&D) include both capital and current expenditures for basic research and experimental development. Physicians (PHY): the number of general and specialist medical practitioners per 1000 population. GDP per capita (GDPP) is gross domestic product per capita and it is proxy for economic growth. Education (EDU) is defined as a percentage of population aged 15 and over that have attained or completed secondary education.

### 2.4. The Data Envelopment Analysis

In 1951, Koopmans first introduced the idea of technical efficiency, according to which a company is technically productive, unless it is impossible to produce more outputs without using more of any input [38]. This description was further refined by Debreu and Shephard [39,40] in the process of extending their work. Farrell, [41] empirically introduced how cost efficiency can be calculated by categorizing into two components: technological and allocative (price) efficiency. The first is the ability to achieve optimum output from inputs and the second is the capability to use inputs in optimal proportions. These modules were then grouped to overall economic performance, which was later integrated by Charnes, Cooper, and Rhodes [23] by establishing the non-parametric data envelopment analysis technique into the linear programming system.

The models vary mainly in the assumption that the production function exhibits constant or variable returns to scale in efficient measurement orientation. The two most widely used basic data envelopment analysis models include the Banker, Charnes, and Cooper (BCC model) [42] and Charnes, Cooper, and Rhodes (CCR model) [23]. The first model’s performance reflects the overall technical efficiency, which calculates inefficiencies due to the configuration of input-output and the scale of the process. The second model leads to a technical efficiency score that represents strictly managerial inadequate performance. To avoid the option of model orientation, numerous DEA models have been developed, which simultaneously estimate potential input reductions and output expansions. Thus, on the grounds of the Banker, Charnes, and Cooper model, Charnes, Cooper, Golany, Seiford, and Stutz [42,43] proposed the input and output translation-invariant additive model. This model was then expanded by Tone, [44] to the Slacks-based Measurement (SBM) model by unit invariant and monotone efficiency measurements. In addition, a non-oriented model that transforms data using a logarithmic structure was constructed by Charnes, Cooper, Seiford, and Stutz [43].

The Slacks-based Measurement as employed to examine the efficiency scores among the countries studied. Tone [44] recommended SBM of efficiency to the outmoded radial DEA model. However, the slack variables of the SBM model are also directly added to the target function to evade overestimating efficiency. The SBM procedure is non-radial and deals directly with input/output slacks by removing the oriented and radial deviation. The SBM model is provided below.

(2) $$min\rho =\frac{1-\frac{1}{m}\sum_{i=1}^{m}s_{i}^{-}/x_{ik}}{1+\;\frac{1}{q}\;\sum_{r=1}^{q}s_{i}^{+}/y_{rk}\;\;}\;\;\;\;$$

where is subject to

(3) $$\overset{n}{\underset{j=1}{\sum }}y_{ij}\lambda_{j}-s_{i}^{-}=x_{i0}$$

(4) $$\overset{n}{\underset{i=1}{\sum }}y_{rj}\lambda_{j}+\;s_{r}^{+}=\;y_{r0}$$

(5) $$\lambda ,\;S^{-},\;\;\;\;\;\;S^{+\;}\geq 0$$

(6) j=1,2,…,n,i=1, 2,…,m,r=1,2,…,q1, 2…, n,i = 1, 2,…,m,r = 1,2,…,q

where ρ is the health expenditure with 0 < ρ ≤ 1. *x* with *y* is the perceived values of outputs and inputs. $S^{-}$ and $S^{+\;}$denote output plus input slacks for the DMU in evaluation. λ is a weight coefficient of reference decision making unit (DMU). To deal with the limitation of the SBM model, the study used the super-SBM model. Super-SBM manages excessive input along with scarcity in the output. The study employs additive models to deliver a scalar measure regarding all inefficiencies [45]. The super-SBM model presented as:

(7) $$min\delta =\frac{1+\;\frac{1}{m}\;\sum_{i=1}^{m}s_{i}^{-}/x_{ik}}{1-\frac{1}{q}\sum_{i=1}^{q}s_{r}^{+}/y_{rk}}$$

which is subject to:

(8) $$\overset{n}{\underset{j=1,j\neq k}{\sum }}x_{ij}\lambda_{j}-s_{i}^{-}\leq x_{ik}$$

(9) $$\overset{n}{\underset{j\;=1,j\neq k}{\sum }}y_{ij}\lambda_{j}-s_{i}^{-}\geq y_{rk}$$

(10) $$\lambda ,s^{-},s^{+}\geq 0$$

(11) j=1, 2,…,n, i=1, 2, …,m,r=1, 2, …, q

The super SBM model postulates a constant return of Scale (CRS). This study spread the SBM and super SBM models to the VRS case by restricting $\overset{n}{\underset{j=1}{\sum }}\lambda_{j}=1$ for Equation (2) and $\overset{n}{\underset{j\;=1,j\neq k}{\sum }}\lambda_{j}=1\;$for Equation (11), respectively.

### 2.5. Econometrics Method for the Second-Stage

#### Models Specification

Following the studies of McDonald, [46] the Tobit regression is employed to examine the factors influencing health efficiency within the emerging economies and in estimating the linkages between dependent variable $y_{i}$ (efficiency scores) and a vector of explanatory variables $x_{i}$ [44]. For the *ith* DMU, the Tobit model is mathematically defined as follows.

(12) $$\;y_{i\;}^{*}=x_{i}\beta +\varepsilon_{i}$$

(13) $$\mathrm{If}\;Y_{I}^{*}\leq 0,\;Y_{i},\;Y\_i=0;\;\mathrm{if}\;Y_{I}^{*}\geq 1,\;and\;if\;0<1,\;Y_{I}=\;Y_{I}^{*}$$

where, $y_{i\;}^{*}$ is an observed latent variable, $\varepsilon_{i}$ is identical, normal, and independently distributed with zero variance σ and mean. $x_{i}$ is a vector of the explanatory variables and β is a vector of unknown coefficients. The variables were transformed into logarithmic form.

(14) $$hlteff_{it}=\alpha_{i}+\beta_{1}lnR\&D_{it}+\beta_{2}lnPHY_{it}+\beta_{3}lnCRP_{it}+\beta_{4}lnGDPP_{it}+\;\beta_{5}lnEDU_{it}+\mu_{it}$$

(15) $$hlteff_{it}=\alpha_{i}+\beta_{1}lnPHY*lnR\&D_{it}+\beta_{2}lnCRP_{it}+\beta ln_{3}GDPP_{it}+\;\beta_{4}lnEDU_{it}+\;\varepsilon_{it}$$

where, *i* and *t* represent country and time while *µ* and *ε* represent the error term, respectively. The study again employed the Simar and Wilson estimation method for a robust check to examine the influence of dependent variables on health efficiency. A detailed explanation of the study variables is presented in Table 2 below.

## 3. Results

### 3.1. Descriptive Analysis

The mean, median, maximum, minimum, coefficients of variation, standard deviation (SD), probability, and Jarque-Bera estimations were performed in the descriptive analysis. Evidence from Table 3 denotes research and development (R&D) as the variable with the highest mean and standard deviation of 21.064 and 1.508 US$, respectively, indicating R&D as a critical variable in the study. R&D again is the variable with the highest maximum value of 24.170 US$. The coefficient of variations (CV) of the study variables designates the variances within the variables with corruption showing the highest variance of 34.164 among the variables.

### 3.2. Stage 1. DEA Efficiency Scores among the Selected Emerging Nations

The findings in Table 4 reflect that upper middle-income and high-income countries such as Thailand, China, Russia, Greece, and Venezuela are also inefficient. Surprisingly, other lower-income countries such as India with the second-largest population in the world with little resources, could utilize its health resources efficiently. However, other middle-income countries proved not to be health efficient.

**Figure 2:**
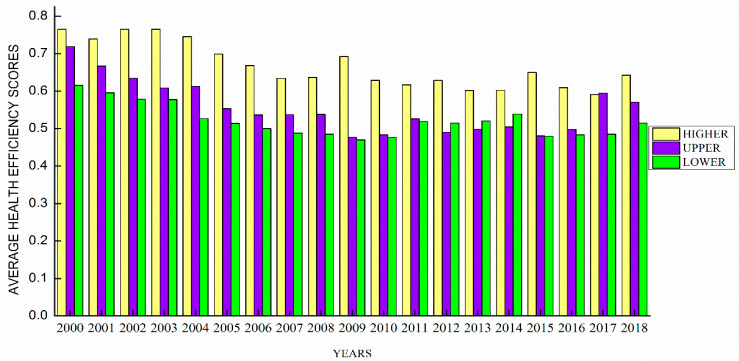
Average efficiency scores for health efficiency.

Figure 2 mirrors the DEA’s efficiency scores among the high, upper, and lower-income countries within the emerging economies. It can be observed that the higher income economies are the star performers following the upper and lower middle-income economies.

### 3.3. Stage 2. Econometric Results

After calculating for health efficiency scores, the study examined the factors influencing healthcare efficiency. The Tobit regression and the Simar Wilson bootstrap estimation method is used to confirm the findings.

#### 3.3.1. Tobit Regression Results

Two models were employed in the study. Model (1) examines the direct relationship between health efficiency and research and development, number of physicians, corruption, income, and education. The next model considers the interactive effect of physicians and research and development on health efficiency. This is very important because findings from the study would provide theoretical knowledge of how these factors function together to enhance healthcare efficiency within the emerging nations.

Model 1 results in Table 5 show that research and development (R&D) is significantly positive in the main panel, upper middle income, and high-income countries. This indicates that a unit rise in R&D leads to an upsurge in health efficiency to about 0.459%, 0.0673%, and 0.087% for the main panel, upper income, and high income, respectively. However, it is negatively significant for lower-middle-income countries. This indicates that 1% upturns in R&D decrease efficiency to about 0.264%.

In addition, economic growth showed positively significant increases in all the panels. An increase in economic growth could result in an upturn in efficiency to about 0.168%, 0.84%, 0.117%, and 0.303% for the main, lower, upper, and high incomes, respectively. Education raises health efficiency in the main panel, lower-middle, and high-income countries. A unit increase in education leads to about 0.0711%, 0.134%, and 0.0538% for the main, lower-middle, and high-income countries. Moreover, physicians increase health efficiency in upper-middle and high-income economies while reducing healthcare efficiency in lower-income nations. The implication is that an increase in the number of physicians could lead to a rise in about 0.391% and 0.00643% for upper-middle and high-income countries, respectively. It may also reduce health efficiency in the lower-middle-income countries by 0.078%. In addition, corruption decreases health efficiency in the main panel, upper middle, and lower middle-income economies but only significant for the main panel and lower middle-income nations. This suggests that a unit escalation of corruption results in a decline in health efficiency to about 0.115% and 0.026% for the main panel and lower middle-income countries.

Model 2 results for Tobit in Table 6 demonstrate that economic growth is positively significant. An indication that a 1% increase in economic growth raises health efficiency to about 0.130% for the main panel, 0.290% upper-middle-income, and 0.0661% high middle-income countries. Again, education is positive and increases health efficiency in the main panel, upper-middle, and high-income panel to about 0.0821%, 0.143%, and 0.196%, respectively. The interaction effect between R & D and physicians is positively significant in the main, lower-middle, upper-middle, and high income. This implies that a unit upsurge in the interaction term leads to upturns in health efficiency to about 0.0631%, 0.00248%, 0.134%, and 0.099% for the main, lower-middle, upper-middle, and high-income levels, respectively. Furthermore, corruption reduces health efficiency across all countries and is only statistically negative and significant for the main and lower-middle-income countries.

#### 3.3.2. Simar and Wilson Regression Analysis

The results in Table 7 for the Simar and Wilson efficiency analysis is similar to the Tobit regression results presented in the above models in Table 5 and Table 6. The coefficients are largely different but the signs in both Simar-Wilson and Tobit regression are quite similar. The coefficient for per capita GDP is positive and significant across all panels. Again, R&D is positive and increases healthcare efficiency in the main panel, high income, and lower income panel. Physician’s coefficient is positively significant and increases health efficiency in the main, high income, upper middle income, and lower middle-income panel. With respect to corruption, it reduces health efficiency in the middle-income and lower-income countries while the other income countries remain insignificant.

Simar and Wilson estimation results for model 2 in Table 8 indicate the coefficients and the signs are similar to the Tobit results. All the variables including, per capita GDP and education, are positively significant and increase health efficiency while corruption showed a negative relationship with healthcare efficiency. In the case of lower middle-income nations, income has a negative relationship. The interaction effect between physicians and research and development promotes health efficiency at all panel levels except lower middle income where it positive but insignificant. The Simar and Wilson estimation procedure, therefore, serves as a robust check for our analysis. Since the results in both models and estimations techniques are similar, the findings are robust and reliable for policy suggestions.

## 4. Discussion

There is growing pressure on health decision-makers to provide measures that improve population health. To investigate the factors affecting healthcare efficiency within the selected emerging nations, the first stage of the study compared health efficiency scores among the study countries of mainly lower income, upper middle-income, and high-income levels. Analysis of the study from stage 1 using the Slacks-based Measurement Data envelopment analysis (DEA) model revealed that other lower-income countries like India and Bulgaria are productive in utilizing inputs within the healthcare system. In contrast, other middle-income countries proved to be health inefficient. A reason could be that these middle-income countries were complacent about their past success and, therefore, and continuously ignore improving the utilization of healthcare resources, the result is inefficiency.

Given this, the study moved further to examine the determinants of health efficiency within the emerging nations using the Tobit regression and the Simar Wilson estimation procedure. The study’s outcome revealed that medical research played an irreplaceable role among the main panel, upper middle income, and high-income countries in emerging nations. This indicates that R&D is very vital in many sectors of the economy, including the health sector. Medical research could lead to improved medicines and cure of diseases. An increase in health care research may also help expand the knowledge of health professionals, eliminate, guess work, and profoundly understand the principles underlying certain health actions. An upsurge in medical research can also enhance the ability to predict possible outcomes of physicians’ decisions. For instance, Herceptin’s success and other therapies for breast cancer and coronavirus diseases are classic examples of the importance of health research [50], which is a substantial improvement in public health. However, in the lower-middle-income countries, research and development spending remained insignificant to health efficiency. The implication of this finding may be related to other factors such as socio-economic and cultural barriers that can potentially influence the lower-income nation’s willingness to participate in health research [51].

Economic growth promotes health efficiency in the main panel, lower middle income, and high-income countries. The implication can be that, as countries develop economically, they are able to allocate health resources that can be used for health services. Similarly, as the economy grows, governments may provide adequate compensation and favorable working and living conditions for healthcare providers, leading to job satisfaction and greater health efficiency. The study concedes with concrete evidence from Subramanyam and Kawachi et al. [52] in India and Cole [53] in developing economies. However, in the upper-middle-income countries, income reduces health efficiency. The reason can be that, as income increases in upper-middle-income countries, not enough of the percentage of the GDP is invested in the health sector. When this happens, governments may be unable to deliver sufficient resources for health services leading to inefficiencies.

Education is also conducive to health efficiency in the main panel, upper middle income, and high-income countries. Again, people with educational background can acquire health information and comply with medical treatment that could result in increased health efficiency in these countries. Educated persons can appreciate their health needs, communicate well with health professionals, and advocate for themselves and close relatives for a better health outcome [54].

Moreover, a surge in the number of physicians increase health efficiency in the main panel, lower middle income, and high-income economies. The ratio of physician to population affects the health status of the people in the country. This shows that, when the number of physicians increases, it enhances patients’ chances of being diagnosed and treated during the early stages of their disease condition [55]. Our study suggests that an increase in the number of physicians in emerging countries may assist patients to minimize pain and recover from a disease faster. They also help patients modify their risky behaviors on health and safe practices [55]. Conversely, physicians reduce health efficiency in the upper middle income. This result could be related to the ratio of physician to the population in the health sector because the number of patients per physicians may be high, and, therefore, reduces the contact hours for providing medical care to patients.

Additionally, corruption has an enormous negative effect on health in all the panels. This indicates that corruption reduces health efficiency in emerging economies. Corruption negatively influences public health care policies by hindering physicians and health workers from providing services to the optimal level due to inadequate resources. Corruption is found to reduce health efficiency because some health providers can divert health resources purposed for effective health delivery for their gains. In another view, corruption in emerging economies could also reduce government revenue for health, which sequentially reduces the quality and quantity of health service as a low tax revenue is attributed to fewer governments’ health expenses. The study is in harmony with the findings of Lawrence [56] and Dincer and Teoman [57] which also confirmed the negative effect of corruption on the efficiency of healthcare.

## 5. Conclusions

Good health is a crucial rudiment of public health and a vital health-related parameter. The study aimed to compare health efficiency and its contributing factor among the emerging nations. Data was obtained from the World Bank’s World Development Indicators for 21 emerging countries from 2000 to 2018. Two-stage Data Envelopment Analysis (DEA) was employed for the analysis. The first stage utilized the Slacks-based Measurement model to compute health efficiency scores. The results from the DEA analysis showed the potential of lower-income countries in optimizing their health resources. The second stage investigates the contributing factors of health efficiency utilizing Tobit regression and Simar-Wilson for a robust check.

The Tobit robust to Simar-Wilson results denotes that medical research and development is significantly positive in the main panel, upper middle income, and high-income countries. Economic growth increases health efficiency in all the panels. Education raises health efficiency in the main panel, lower-middle, and high-income countries. Additionally, corruption decreases health efficiency in the main panel and lower middle-income nations. The interaction between R&D and physicians enhances health efficiency in all the panels.

The following recommendations are, therefore, suggested based on the findings.

Corruption reduces health efficiency in most study panels indicating its detrimental consequences on health delivery in the emerging countries. Electronic payment can be introduced for monetary transactions to minimize corruption in the health sector. Again, consistent monitoring is a good sign for countries that aspire to do away with corruption. Governments in, emerging economies are encouraged to adopt a disciplinary strategy that would help governments establish stricter laws and regulations, especially in the health sector. Again, culprits should be severely punished and should be made to bring back or pay for whatever they have benefited from unlawfully according to the country’s laws and constitution to deter others. It is hoped that, when the laws are firmly obeyed, it will help create a conducive environment for monitoring and accountability, helping reduce corruption. In addition, trackers could be placed on some of the easily movable items in hospitals for easy retrieval if it is misplaced. Moreover, education improves health efficiency in most of the panels. Education should be improved by providing the necessary infrastructure because it promotes economic development, and increases health efficiency. The interactive effects between physicians and research development enhance health efficiency. It is, therefore, important for all studied countries to train more physicians and improve medical research at all levels.

### Future Direction

In subsequent studies, it is useful to examine the influence of other environmental factors in African countries to better understand how they impact health expenditure in these countries. Although the DEA method is being adopted in this study, it would be useful to include sensitivity analysis involving stochastic frontier analysis in future studies. However, we hope this study will initiate other attempts of similar direction in other regions by providing useful information for policy-makers in drafting health policies in the future.

## Figures and Tables

**Table 1:** Classification of emerging nations.

Group	Country
High-income	Czech Republic, Greece, Argentina, and Chile
Upper-middle income	Mexico, Turkey, Brazil, Thailand, Brazil, China, Russia, South Africa, Peru, Russia, United Arab, Emirates, Poland, Bulgaria, and Venezuela.
Lower-middle income	India, Bangladesh, Philippines, Indonesia, India, Ukraine, and Pakistan.

**Table 2:** Determinants of health efficiency.

Acronym	Variable Name	Units	Source
CRP	Corruption (CRP)	1-low to 6- high of which countries with least score represent higher corruption level and the vice versa	International Country Risk Guide (ICRG), 2019 [47]
R&D	Research and Development expenditure	Percentage (%) of GDP	WDI, 2019 [48]
PHY	Number of physicians	Physicians per 1000 population	WDI, 2019 [48]
EDU	Education	Population aged 15-64 who have completed	Barro and Lee 2019 [49]
GDPP	Gross Domestic Product per capita	Current US $	WDI, 2019 [48]

**Table 3:** Descriptive statistics.

Statistics	CRP	lnEDU	lnGDPP	lnPHY	lnR&D
Mean	2.33	13.048	8.586	11.399	21.064
Median	2.416	12.790	8.838	11.064	20.825
Maximum	5	21.084	11.901	14.843	24.170
Minimum	1	10.899	6.263	9.528	18.338
Std. Dev.	0.797	1.3941	0.994	1.386	1.508
Skewness	0.734	1.093	0.625	0.843	0.370
Kurtosis	4.117	5.705	2.901	2.643	2.141
Jarque-Bera	45.605	161.920	21.017	39.753	17.171
Probability	0.000	0.000	0.000	0.000	0.000
CV	34.164	10.684	11.575	12.160	7.158

In estimating the convergence between the countries studied, the coefficients of variance (CV) are computed as standard (deviation/mean × 100). lnGDPP, lnEDU, lnPHY, CRP represent log of research and development, income, education, physicians and corruption accordingly.

**Table 4:** Health efficiency scores for the selected emerging economies.

Country	Years																		
	2000	2001	2002	2003	2004	2005	2006	2007	2008	2009	2010	2011	2012	2013	2014	2015	2016	2017	2018
Pakistan	0.296	0.271	0.295	0.418	0.381	0.363	0.319	0.283	0.271	0.463	0.293	0.287	0.317	0.324	0.323	0.371	0.389	0.359	0.344
India	0.941	0.985	0.876	1.000	1.000	0.771	0.687	0.667	0.694	0.558	0.744	0.809	0.753	0.709	0.661	0.644	0.620	0.597	0.572
Bangladesh	0.247	0.292	0.299	0.294	0.237	0.230	0.221	0.231	0.231	0.247	0.251	0.300	0.294	0.304	0.295	0.305	0.308	0.323	0.161
Philippines	0.229	0.209	0.188	0.180	0.175	0.162	0.154	0.159	0.143	0.126	0.132	0.145	0.144	0.148	0.542	0.429	0.532	0.628	0.546
Colombia	0.731	0.738	0.865	1.000	0.679	0.578	0.512	0.452	0.399	0.415	0.429	0.450	0.445	0.439	0.390	0.376	0.376	0.370	0.581
Brazil	0.901	0.868	0.844	1.000	0.834	0.820	0.680	0.615	0.562	0.523	0.502	0.497	0.465	0.462	0.462	0.439	0.417	0.400	0.568
Thailand	0.851	0.858	0.882	0.865	0.856	0.841	1.000	1.000	0.987	0.921	0.964	1.000	0.919	1.000	1.000	0.981	1.000	0.932	1.000
China	1.000	1.000	0.916	0.943	0.916	0.870	0.897	1.000	1.000	1.000	1.000	0.941	0.970	0.950	1.000	0.931	0.953	1.000	1.000
South Africa	0.390	0.408	0.420	0.432	0.394	0.387	0.400	0.423	0.401	0.416	0.416	0.402	0.389	0.381	1.000	0.351	0.335	0.345	0.064
Peru	0.444	0.431	0.415	0.400	0.409	0.394	0.400	0.388	0.379	0.370	0.360	0.344	0.333	0.331	0.393	0.491	0.604	1.000	0.906
Czech Republic	1.000	0.721	0.693	0.574	0.545	0.497	0.466	0.401	0.328	0.315	0.333	0.347	0.349	0.339	0.386	0.349	0.404	0.616	0.424
Russia	0.661	0.570	0.478	0.385	0.199	0.225	0.282	0.258	0.398	0.521	0.607	0.745	0.763	1.000	1.000	0.874	1.000	0.985	1.000
United Arab Emirates	0.578	0.646	0.416	0.412	0.412	0.438	0.434	0.397	0.403	0.398	0.389	0.388	0.383	0.366	0.345	0.335	0.328	0.318	0.477
Poland	0.367	0.360	0.350	0.350	0.326	0.335	0.315	0.294	0.279	0.262	0.269	0.575	0.560	0.550	0.284	0.260	0.269	0.265	0.334
Ukraine	0.476	0.428	0.403	0.351	0.330	0.313	0.295	0.292	0.302	0.311	0.311	0.297	0.279	0.275	0.297	0.318	0.316	0.298	0.265
Bulgaria	0.800	0.832	0.771	0.727	0.696	0.646	0.599	0.581	0.524	0.501	0.462	0.439	0.519	0.679	0.747	0.636	0.731	0.828	0.606
Venezuela	1.000	0.899	0.886	0.887	0.875	1.000	0.921	0.919	1.000	0.727	0.759	1.000	1.000	1.000	1.000	1.000	0.859	0.824	1.000
Malaysia	0.665	0.796	0.785	0.663	0.597	0.559	0.504	0.474	0.466	0.438	0.409	0.397	0.671	0.750	0.891	0.779	0.577	0.668	0.655
Greece	1.000	0.823	0.749	0.733	0.759	0.723	0.664	0.746	0.706	0.670	0.590	0.542	0.520	0.597	0.540	0.477	0.542	0.685	0.608
Turkey	0.368	0.368	0.368	0.368	0.368	0.368	0.368	0.368	0.368	0.368	0.368	0.368	0.368	0.368	0.368	0.368	0.368	0.368	0.368
Argentina	0.476	0.428	0.403	0.351	0.330	0.313	0.295	0.292	0.302	0.311	0.311	0.297	0.279	0.275	0.297	0.318	0.316	0.298	0.265

**Table 5:** Model 1: Tobit regression.

Variable	Panel	Lower	Upper	High
lnR&D	0.459 *	0.84 **	0.0673 **	0.03
	(0.248)	(0.032)	(0.0292)	(0.07)
lnGDPP	0.168 ***	0.84 *	0.117***	0.303 *
	(0.025)	(0.32)	(0.035)	(0.156)
lnEDU	0.0711 ***	0.134 ***	0.03	0.0538 **
	(0.0259)	(0.048)	(0.03)	(0.0205)
lnPHY	0.0426	−0.0778 **	0.391 ***	0.0064 ***
	(0.026)	(0.034)	(0.035)	(0.001)
CRP	−0.115 **	−0.0337 **	−0.033	0.026
	(0.101)	(0.027)	(0.04)	(0.020)
Constant	−0.661 **	1.199 ***	1.300 *	3.601
	(0.318)	(0.359)	(0.66)	(2.357)
Chi square	57.55	96.52	71.33	66.9
Prob > chi2	0.000	0.000	0.000	0.000
Pseudo R2	0.1985	3.9129	0.4642	1.8331

Standard deviations are in parenthesis. ***, **, * represents 1%, 5%, and 10% significance level. lnR&D, lnGDPP, lnEDU, lnPHY, CRP represent log of research and development, income, education, physicians and corruption accordingly.

**Table 6:** Model 2: Tobit regression.

Variable	Panel	Lower	Upper	High
lnGDPP	0.130 ***	0.0576	0.0661 ***	0.290 ***
	(0.0241)	(0.07)	(0.021)	(0.049)
lnEDU	0.0821 ***	0.06 *	−0.05	0.196 **
	(0.0264)	(0.03)	(0.10)	(0.0934)
lnPHY*lnR&D	0.0631 ***	0.00248 ***	0.134 ***	0.099 ***
	(0.0235)	(0.00086)	(0.048)	(0.041)
CRP	0.0778 **	−0.171 **	−0.0102	−0.0449
	(0.0342)	(0.0733)	(0.0407)	(0.0408)
Constant	−1.347 ***	−0.366	0.467	−0.462
	(0.376)	(0.389)	(0.865)	(1.587)
Chi square	43.92	31.48	61.74	48.21
Prob	0.000	0.000	0.000	0.000
Pseudo R^2^	0.1514	1.276	0.4018	1.3208

Standard deviations are in parenthesis. ***, **, * represents 1%, 5%, and 10% significance level. lnGDPP, lnEDU, lnPHY*lnR&D, CRP represent log of research and development, income, education, interactive effect of physicians and research and development, and corruption accordingly.

**Table 7:** Model 1: Simar and Wilson regression.

Independent Variable	Panel	Lower	Upper	High Income
lnGDPP	2.0214 ***	0.147 ***	0.720 **	1.621 *
	(0.042)	(0.024)	(0.125)	(0.451)
lnR&D	0.050 ***	0.113 ***	2.001	0.0750 ***
	(0.011)	(0.037)	(0.991)	(0.0279)
lnEDU	1.113 ***	−0.901	0.974 ***	0.321
	(0.001)	(0.654)	(0.084)	(0.125)
lnPHY	0.421 ***	0.512 *	3.051 ***	1.215 ***
	(0.021)	(0.041)	(1.254)	(0.051)
CRP	0.0453	−0.173 ***	−0.0492	−0.147
	(0.041)	(0.000)	(0.0474)	(0.104)
Constant	0.238 **	0.253 **	0.696 ***	1.074 ***
	(0.114)	(0.118)	(0.132)	(0.223)

Standard deviations are in parenthesis. ***, **, * represents a 1%, 5%, and 10% significance level lnR&D, lnGDPP, lnEDU, lnPHY, CRP represent log of research and development, income, education, physicians and corruption accordingly.

**Table 8:** Model 2: Simar and Wilson regression.

Independent Variable	Panel	Lower	Upper	High
lnGDPP	0.083 *	0.397 ***	0.570 **	−0.05
	(0.001)	(0.053)	(0.005)	(0.13)
lnPHY*lnR&D	2.150 ***	3.051 ***	0.193 **	0.541
	(1.002)	(1.254)	(0.096)	(0.000)
lnEDU	0.397 ***	0.199 *	0.208	0.193 **
	(0.053)	(0.113)	(0.20)	(0.096)
CRP	−0.171 **	−0.201 **	−0.028	0.019
	(0.773)	(0.243)	(0.224)	(0.021)
Constant	0.581 ***	1.030 ***	1.155 ***	1.185 ***
	(0.0612)	(0.113)	(0.204)	(0.104)

Standard deviations are in parenthesis. ***, **, * represents a 1%, 5%, and 10% significance level. lnR&D, lnGDPP, lnEDU, lnPHY, CRP represent log of research and development, income, education, physicians and corruption accordingly.

## Data Availability

The data used to support the findings and conclusions of this study was extracted from World Bank World Development Indicator WDI (2019) under license and copyright of World Bank World De-velopment Indicators. Requests for access to these data can be made to World Bank World De-velopment Indicator WDI (2019) http://datatopics.worldbank.org/world-development-indicators/.
